# “Expected to happen”: perspectives on post-release overdose from recently incarcerated people with opioid use disorder

**DOI:** 10.1186/s12954-024-01055-1

**Published:** 2024-07-22

**Authors:** Pryce S. Michener, Elyse Bianchet, Shannon Fox, Elizabeth A. Evans, Peter D. Friedmann

**Affiliations:** 1 Population Health Sciences, Graduate School of Biomedical Sciences, University of Massachusetts Chan Medical School, 55 N Lake Ave, Worcester, MA 01655 USA; 2 Dept. of Medicine, UMass Chan Medical School – Baystate, 759 Chestnut St, Springfield, MA 01199 USA; 3 Tufts Medical School, 145 Harrison Ave, Boston, MA 02111 USA; 4 Dept. of Health Promotion and Policy, School of Public Health and Health Sciences, University of Massachusetts Amherst, 312 Arnold House, 715 North Pleasant Street, 01003 Amherst, MA USA

**Keywords:** MOUD, Opioid use disorder, Risk environment framework, Overdose, Qualitative, Incarceration, Jail

## Abstract

**Background:**

Opioid-related overdose is the leading cause of death for people recently released from incarceration, however treatment with medications for opioid use disorder (MOUD) during incarceration can reduce the mortality risk. This study seeks to qualitatively analyze perceptions of post-release overdose risk from the perspectives of people who received MOUD while incarcerated in one of eight Massachusetts jails during 2021–2022 using the Risk Environment Framework to guide analyses.

**Methods:**

*N* = 38 participants with lived experience of MOUD treatment during incarceration who are now living in the community were interviewed on factors that may contribute to or protect against post-release overdose risk. Themes were identified inductively and deductively using the Risk Environment Framework and its domains, which organizes themes along physical, social, economic, and policy environments on both the micro- and macro- scales.

**Results:**

The physical risk environment included loss of opioid tolerance during incarceration, polysubstance use, and the toxicity of the regional drug supply as key producers of increased risk for post-release overdose. Social drivers of risk included peer group risk norms—including peer-driven harm reduction practices and interpersonal relationships between drug sellers and buyers—as well as macro-level social determinants of health such as housing insecurity and availability of mental health services. Economic drivers of post-release overdose risk included lack of income generation during incarceration and employment challenges. Participants discussed several aspects of policy that contribute to post-release overdose risk, including availability of harm reduction supplies, public health services, and broader policy around MOUD.

**Conclusions:**

The perspectives of people with lived experience are vital to understanding the disproportionate risks of overdose for those recently released from incarceration. Our results highlight the intersectional factors that produce and reproduce the post-release overdose risk environment, providing support for interventions across each domain of the Risk Environment Framework. By capturing perspectives from people with lived experience of OUD and incarceration during this critical period of risk, we can better identify interventions that target and mitigate overdose-related harm in this population.

## Introduction

Opioid-related overdose is the leading cause of death for people recently released from jail or prison, with an estimated 120-fold greater risk of overdose death compared to the non-incarcerated population in Massachusetts [1–3]. Although treatment with medications for opioid use disorder (MOUD) such as methadone and buprenorphine during incarceration has been shown to reduce the likelihood of overdose death for people recently released [4–6], overdoses remain high, highlighting the necessity for further investigation into and interventions to address risk factors for overdose after release from incarceration.

In 2019, the state of Massachusetts (MA) mandated that seven state jails begin offering all federally-approved MOUD treatment options as part of a pilot program (Chap. 208). Prior to this legislation, several jails had pre-existing programs for buprenorphine and/or naltrexone treatment for certain populations of incarcerated people with OUD, however no jails provided access to methadone until 2019. The Massachusetts Justice Community Opioid Innovation Network (JCOIN) was funded to conduct a Type 1 hybrid effectiveness- implementation study of Chap. 208 [7]. As part of the JCOIN study, qualitative interviews were conducted with people who were incarcerated in one of the seven jails who participated in the pilot program and received MOUD while incarcerated. These interviews offer a unique opportunity to understand how post-release overdose risk may be modified by MOUD treatment during incarceration from the perspectives of people with lived experience. By highlighting the voices of people who are directly impacted by the implementation of MOUD in MA jails, we can better understand how the risk environment of post-release overdose is experienced day-to-day, and how these risks may be identified, addressed, and mitigated. Additionally, understanding patient perspectives of post-release overdose risk will allow for more informed, culturally competent, and targeted education and other interventions to address this critical public health issue.

A conceptual model of post-release overdose risk has identified how exposure to incarceration impacts overdose risk through several mechanisms including disruptions in social support networks, mental health, interruptions in medical care, stigma, and increased likelihood of risky drug use behaviors [8]. The risk environment framework takes a broader view, positing that drug-related health risks are products of the interaction between individuals and their environments, categorizing risk as relating to physical, social, economic, and policy domains embedded in the micro- and macro- environment [9, 10]. The micro-environment is defined as the individual factors and immediate context of drug use that result in risk production, for example interpersonal relationships with peers who use drugs, the physical location where drugs are consumed, and local availability of harm reduction resources such as sterile injection supplies. The macro-environment is defined as large-scale systemic factors which interact with the micro-environment to (re)produce risk, such as drug trade routes, broader societal stigma against people who use drugs, law enforcement practices, and public health policy [9, 10].

Although categories are presented as discrete, the framework acknowledges the intersections and relationships between different domains [9]. The risk environment framework is particularly useful for understanding post-release overdose risk as it allows for the analysis of the complex interactions between carceral and societal institutions and the people who experience these systems through the lens of physical, social, economic, and political environments. The risk environment framework has been previously utilized to assess how rural environments in the United States impact overdose risk [11–14], although to our knowledge, the risk environment framework has not yet been utilized to assess post-release overdose risk.

The perspectives of people who have lived experience with opioid use disorder (OUD), MOUD treatment during incarceration, and post-release overdose are critical to fully understand post-release overdose risk, validate or modify the conceptual models, and develop effective interventions [15–18]. Therefore, we aim to understand the perspectives of people with lived experience and analyze factors that may be protective and contributive to post-release overdose risk in this population.

## Methods

### Participants

Participants were recruited from the eight county jails in Eastern and Western Massachusetts who offer MOUD to incarcerated individuals, including the seven original adopters of MOUD programs and one additional jail which implemented MOUD at a later date. The counties were in urban, suburban, and rural areas and offered varying forms of FDA-approved MOUD. Interviews were conducted with 38 participants of the MOUD programs who were released from incarceration after September 1, 2019 and currently living in the community. Recruitment efforts included posting informational flyers in community locations where previously incarcerated individuals were known to frequent such as treatment clinics and sober housing. The same flyers were also included in the jail’s release packets, and jail employees involved with the MOUD population spoke to clients about the interviews. The flyers instructed interested individuals to reach out to the research team for more information. In addition, some counties received permission from individuals to contact them post-release, and these individuals were contacted directly about the study.

### Data collection

From Fall 2021-Fall 2022, semi-structured phone interviews lasting 30–60 min were conducted with participants. Participants provided verbal consent at the start of each interview and were compensated $40 for their time. The interviewer team consisted of males and females who are an anthropologist, public health doctoral candidate, social worker, clinical psychologist, and master’s level staff members. All interviewers were given group and individual training sessions to ensure consistency across the team. The Exploration, Preparation, Implementation, and Sustainment (EPIS) framework for implementation of public service programs informed construction of the interview guide [19]. The interview guide highlighted the Implementation and Sustainment domains of the EPIS framework and solicited participants’ perspectives on: experiences with MOUD both in jail and after release into the community, factors that facilitate and impede MOUD delivery in these settings, reentry planning, risk of overdose after release from incarceration, stigma, and recommendations for future MOUD implementation efforts. The interviews were digitally recorded then transcribed and redacted. The Baystate Health Institutional Review Board approved all study procedures.

### Data analysis

A coding team consisting of four staff members created a codebook based off the questions in the interview guide. Additional codes were added as other themes emerged after initial transcript review. The final code book consisted of 23 parent codes and 32 child codes, which was finalized by utilizing open coding and constant comparative methods. All four coders coded the same first four transcripts in order to review code applications and refine code definitions as necessary. Moving forward, the coders worked in dyads, coding each transcript on their own then meeting as a pair to compare codes and review questions. Coded transcripts were uploaded and analyzed through Dedoose v9 [20]. In related work, these data have been analyzed to examine client perceptions of MOUD diversion in jails [21] with additional manuscripts in progress analyzing client perspectives on extended release buprenorphine, community reentry, and perceptions of jail-based MOUD treatment [22].

This manuscript summarizes thematic findings from two codes: overdose and return-to-use following release from jail. Analysts employed inductive and deductive coding strategies using the risk environment framework and its domains. Data were reviewed independently by each analyst and emergent themes were derived using a data-driven thematic coding scheme in keeping with grounded theory [23, 24]. Analysts compared summaries of emergent themes and utilized the risk environment framework [9, 10] as a lens to understand how overdose risk is produced and impacted by social, physical, political, and economic contexts. Colloquialisms and utterances were removed from quotes to improve readability.

## Results

Participants had a mean age of 41.5, and a majority were male, white, and non-Hispanic/Latinx (Table 1). Most (79%) of participants had attained a high school diploma or equivalent or higher educational degree. Buprenorphine (daily) was the most common post-release MOUD among participants (55.6% of those receiving MOUD) followed by methadone (30.6% of those receiving MOUD). Two participants were not receiving MOUD at the time of the interview. The number of days between participants’ release from incarceration and the interview ranged from 3 to 768 days with a median of 51.5 days. Approximately a third of participants had been living in the community for fewer than 30 days post-release at the time of the interview. Qualitative results are presented according to the domains of the risk environment framework (Table 2).

**Table 1:** Characteristics of participants (*n* = 38)

Characteristic	Count (%)
**Age**, mean (SD)	41.5 (9.3) Missing = 1
**Female**, n (%)	4 (14.3%)
**Race**, n (%)	
Native Hawaiian or Other Pacific Islander Black or African American White More than one race	1 (2.6%) 3 (7.9%) 32 (84.2%) 2 (5.3%)
**Hispanic/Latinx ethnicity**, n (%)	9 (23.7%)
**Education**, n (%)	
No high school diploma High school diploma or equivalent Some college, but no degree Associate’s degree	8 (21.1%) 19 (50.0%) 9 (23.7%) 2 (5.3%)
**Not currently taking MOUD**, n (%) **Currently taking MOUD**, n (%) Buprenorphine (e.g., Suboxone, Subutex) XR-Bup (Sublocade) XR-Naltrexone (Vivitrol) Methadone	2 (5.3%) 36 (94.7%) 20 (55.6%) 4 (11.1%) 1 (2.8%) 11 (30.6%)
**Release to interview interval**, n (%)	
0–30 days 31–60 days 61–90 days 91–120 days > 120 days	13 (34.2%) 9 (23.7%) 2 (5.3%) 0 (0.0%) 14 (36.8%)
**County of incarceration**, n (%)	
A B C D E F G H	1 (2.6%) 6 (13.2%) 4 (10.5%) 8 (21.1%) 3 (7.9%) 6 (15.8%) 3 (7.9%) 7 (18.4%)

**Table 2:** Summary of key qualitative themes according to Risk Environment Framework domains (adapted from Rhodes, 2002)

	Micro-environment	Macro-environment
**Physical**	Drug injecting practices Tolerance loss during incarceration Underdosing of MOUD Polysubstance use	Toxic drug supply High prevalence of fentanyl Location of recovery services and proximity to areas of high drug use
**Social**	Social and peer group risk norms Social support networks Peer-driven harm reduction	Lack of accessible housing Community support networks Low availability of mental healthcare Community experience with overdose
**Economic**	Lack of income generation during incarceration Community reentry support	Employment stigma against people with history of criminal-legal system involvement
**Policy**	Availability of harm reduction materials (sterile injection supplies, naloxone, etc.) Implementation of health services	Drug laws Legislative mandate for MOUD provision in MA jails

### Physical micro-environment

#### Tolerance changes

The vast majority of participants described opioid tolerance loss during incarceration as a major driver of post-release overdose risk. Many of these participants noted that it was common for people to return to their pre-incarceration drug use habits following release from jail:

[A]fter being away from the drug for even a short period of time, your tolerance goes way down. It goes way, way down. So if you’re away for two days, you’re at high risk but never mind almost…18 months, you know? So it just really intensifies your use for overdosage and it’s a scary thing. And…there’s not enough awareness about it (ID702).

Most participants tied tolerance loss with unpredictability in the drug supply, factors which combine to exacerbate the high overdose risk following release from jail. Participants reported:

…the number one reason [people overdose] is they try to do the same amount they did before they went in. So it’s like you’re clean…It’s not dope. It’s not heroin. It’s fentanyl…You try to do two bags of fentanyl when you’re clean. And that’s how you overdose because you’re trying to do a little bit too much, too quick…People are always trying to do way too much when they go out, when they get out clean and sober (ID302).

Despite the significant danger posed by these risks, participants reported that receiving MOUD treatment while incarcerated in jail was protective against tolerance loss and post-release overdose. Participants shared personal experiences, with one saying, “I left here before and what saved me before was I was on Suboxone” (ID505). Another compared his own post-release experiences having received medication while incarcerated to when he did not receive medication:

Every time I’ve ever had an experience if I’d gotten home, and I didn’t do any Suboxone, or have any Suboxone in my system before I went home, I went home and [overdosed]…but if I went home, and I had Suboxone in my system…when I did that heroin and that Suboxone, it saved my life… It never let me OD (ID401).

However, an important distinction was made; though receiving MOUD while incarcerated was seen as protective, some noted this protection was limited if a therapeutic dose was not established prior to release:

…being on your medication for an extended period of time reduces the risk of relapsing. They only put you on it three days before you leave, that’s not sufficient enough…that’s what happened to me last time, before I got out, they put me on it literally like two days before I got out, and so it wasn’t enough…and then I just overdose[d], and then I used right when I got out (ID602).

Another participant described the importance of therapeutic dosing for people receiving MOUD who may still be using, or at risk of returning to use, given the context of fentanyl in the drug supply:

I wish they would have raised the dose a little more. Because even at that dose, you still have a risk of overdosing. 50 [mg] methadone, they consider not even a therapeutic blocking dose. I know between like 80 and 120 is considered a blocking dose. So especially with the fentanyl, it’s so strong now that even people on methadone they’re not on the right dose. They’re still experiencing overdoses (ID702).

### Polysubstance use

Beyond tolerance changes, participants described other aspects of personal drug use that contributed to risk of overdose. Namely, polysubstance use with benzodiazepines and alcohol was reported as a driver of post-release overdose, with “mixing other drugs, alcohol, pills,” being mentioned as “the biggest things to [cause] overdose” (ID702).

Participants described how polysubstance use interacts with tolerance loss during incarceration to increase risk of overdose following release, with one respondent saying “People overdose because their tolerance [is] down, or they mix…certain drugs, and they don’t mix. So, if you take benzos with opiates, then you’re at a higher risk for overdose. (ID504). Another participant described how the tendency to increase drug doses and mix opioids with other drugs without accounting for tolerance loss can lead to problems:

…there are some people that like to push the envelope, so they’ll start off small but then they’ll increase quicker that they’re supposed to, and that’s what’s getting them in trouble and on top of that, what they’ll do is they’ll mix it with say a benzo or something like that (ID103).

### Physical macro-environment

#### Toxic drug supply and high fentanyl prevalence

The saturation of fentanyl in the illicit drug supply reported by many participants was inextricably linked to the prevalence of overdose in this population, with one participant saying, “…that’s not dope anymore, there is just fentanyl” (ID303). Another individual described buying heroin as “like playing Russian roulette” (ID305) due to the prevalence of adulterants in the drug supply and the resulting unpredictability of their effects. Fentanyl in the illicit drug supply was noted as a key risk factor for overdose when the presence of fentanyl in heroin or other drugs compounds with tolerance loss during incarceration:

…tolerance is way down and you know the dope out there it…constantly change[s]… it’s getting more and more fentanyl and less and less heroin so it’s just people think they can do the same amount they used to do…and then, they’re dead after that you know…it’s pretty cut and dried that stuff’s killing people left and right nowadays…I’ve lost a lot of friends (ID305).

Others described how the lack of safety in the illicit drug supply led to the perspective that overdose was inevitable or unavoidable, with one participant saying, “…overdosing…nowadays, it just happens. It’s insane how much it happens. It’s almost expected to happen. And it’s like, ‘Oh, you’re going to overdose. Yeah, yeah, yeah’” (ID505).

Due to the ubiquity and perceived inevitability of overdose, fear of overdose was reported to be overall very high among participants. “Heroin ain’t like it used to be,” according to one participant, which prompted them to change their behavior around heroin use: “I’m afraid of it now. It kills people. It kills everybody…I don’t inject. I just take in little bundles” (ID703). Although fentanyl adulteration in the illicit opioid supply was reported as a common cause of overdose, participants also described the perception that fentanyl was present in other illicit drugs, including cocaine and marijuana: “There’s fentanyl in weed, fentanyl in coke…crack with fentanyl” (ID703). Another participant responded that “They’re cutting every drug with it, even Oxycodone has fentanyl in it. Weed has fentanyl in it, you know” (ID506).

Together, these findings illustrate how navigating the illicit drug supply in the era of high fentanyl prevalence creates excessive risk and fear for people who use drugs, especially for those who do not intentionally ingest fentanyl but instead encounter it as an adulterant in non-opioid substances.

### Physical geography

Participants described the post-release physical environment as a contributor to risk of returning to opioid use. One participant highlighted how exposure to drug use was ubiquitous in their area following release from jail:

[F]or me in [CITY 2], everywhere I went there was a lot of active drug use. Especially for people that are coming out of [HOC 7], you have [LOCATION 2] right there. So right into the whole big mess (ID701).

Another participant described how the location of recovery services can position individuals in close proximity to areas of high drug use, which can lead to return to use:

When you transition, you’re going to go into a halfway house like me with 50 other people you do not know in early recovery. Mind you, the crack spots…down the road literally not even a quarter mile away…and most of these houses are like that. They’re…by drug areas because high-class people don’t want these kind of places around them. So, I mean it’s definitely an easy possibility for you to just give up and start using again if you’re not set up the right way (ID403).

At the same time, that individual connected the physical placement of halfway houses to broader societal stigma against people who use drugs, illustrating how the physical and social domains of the risk environment framework can intersect to produce risk.

### Social micro-environment

#### Drug use habits, environment, and harm reduction

Though physical factors such as polysubstance use directly contribute to increased physiological risk of overdose, other elements of personal use were noted as exacerbating factors. Particularly, the social setting was seen as impacting a person’s risk of serious consequences, i.e., whether the person is using alone: “…that would be the biggest reason why people overdose, is that they’re…using alone as soon as they get out of jail without…any type of blocker” (ID103).

Situational awareness when purchasing drugs and personal relationships with drug sellers were also mentioned as an important element in reducing overdose risk. As one participant explained, “You have to be aware of your surroundings and try to be aware of what you’re buying…to be careful of who you buy your stuff from. So, you just can’t buy from anyone cause you don’t know if you’re getting that poison or what” (ID506).

That individual went on to extend the importance of awareness to include one’s social setting—beyond the question of using alone—to the integrity and quality of relationships with others who may be present during use:

[Y]ou just have to be aware of…who you’re being around…can you trust that person? If they’re gonna leave you or not if you overdose, are they gonna try to help you?…Are they gonna administer Narcan, if they’re gonna call an ambulance or something or they’re just gonna drag you out and leave you? (ID506).

Discussions of personal drug use habits in relation to overdose risk were often mentioned alongside and within the context of harm reduction, even where not explicitly identified as such. Techniques mentioned above, such as not using alone and being aware of one’s source and surroundings, are fundamental aspects of harm reduction. One participant, when discussing the dangerousness of the current supply, summarized his advice to reduce overdose risk: “If you are going to use, use with somebody. And have Narcan” (ID404).

### Personal characteristics and choice

Some participants attributed risk of overdose to personal characteristics, particularly willpower, whether explicitly or implicitly, which was then compounded by other risk factors:

I mean, honestly, the only thing that’s going to make anybody relapse…is them…There can be outside influences. There can be things that happen in your life that are going to make you want to do a million things…things that make you want to crash your fucking car into a tree…But it’s all about whether or not you do it (ID304).

Closely tied to willpower, some pointed to personal choice as the ultimate determining factor in an individual’s returning to use post-release, and thus their risk of overdose.

It’s all on the individual…some people are…content with getting high. They don’t know nothing else. And that’s all they want to do. And that’s all they’re gonna continue to do…until they end up overdosing and dying or dying of natural causes. But it sucks, it’s just sad but that’s the truth…. But if the individual wants better for themselves and wants to progress and be a productive member of society, then he’s gonna do everything he has to do in his power to continue to stay on that path (ID101).

Another participant described trying to change peoples’ behaviors around post-release substance use as ultimately futile: “I guess you just can’t change people’s minds with they want to do. I mean, some people are just stubborn, that’s just what they want to do” (ID402). Although the perception that some individuals were resistant to recovery was prevalent among participants in the study, another participant highlighted the difficult internal conflict around returning to use post-release and the role addiction plays in reentry:

With the addiction part of it…it’s hard, you know? When you’re locked up…it’s easy to say, ‘Oh, I’m done. I’m done…I’m not gonna get high no more…’ But the minute you get let go and you’re free and you got all these different choices…some people probably just the addiction’s too strong. You know, they wanna get high. They feel like, ‘Oh, I haven’t got high in a while. Let me get high. I just got out. I’ll just do it this one time and that’s it.’ You know, ‘And I won’t do it no more.’…And it’s that kind of, you know, the addiction starts making the excuses in their head to get high and I think that’s what does it (ID302).

These findings illustrate how participants consider a low level of personal willpower to be a key component of risk for overdose in the post-release period. Willpower is perceived as vital to long-term recovery from addictions, but participants note that the nature of addiction can make it difficult for even the most well-intentioned individuals to resist returning to illicit drug use.

### Social macro-environment

#### Social determinants of health

Social determinants of health including housing insecurity, mental health, and social support networks were reported by participants to impact overdose risk. Multiple participants reported how experiencing homelessness contributed to adverse mental health and feelings of hopelessness, which can lead to riskier personal drug use. One participant described how their experience with homelessness brought them to the point where they no longer cared if the drugs that they were consuming were adulterated with other substances:

I know that I was homeless on the streets for years throughout my life…being with the depression that gets to you, and…not even just depression. Like you just get tired, you know. Like physically, mentally, and emotionally just exhausted…and you’re just using so much at that point…[Y]ou’re not using to get high anymore. You’re not using to do anything but just get through the day…It gets to a point where you just don’t give a fuck anymore, and that’s whatever the fuck you throw in there is whatever you throw in there, and whatever happens, happens, you know (ID304).

One participant described in detail the circumstances that can accumulate and contribute to an individual feeling hopeless and more likely to overdose, and highlighted the importance of assessing those factors prior to release from jail:

…that mental health aspect would be the most important thing…finding out like how deep of a depression they’re actually in because that will tell you how far they’re willing to go to OD…Like a normal person that has a lot to live for might do a tiny little bump, so he feels something but doesn’t go too far with it…And then you have those people that have just burnt every bridge and know that nobody really trusts or respects them anymore and they’ll just go for it, they don’t care. They live, they die – it doesn’t matter. They have no place to go…They have nobody to talk to really. They’re in a halfway house for the hundredth time or they’re in the shelter where they really don’t wanna be and it’s finding those people that just kind of like given up in jail…they’re not even excited to leave jail, because they are steered to the street in the winter like you know what I mean?…I would say those people are very, very, very, very likely to OD (ID103).

Other participants made the direct connection between social determinants of health, such as housing insecurity and lack of social support, and risk for overdose:

I know that when my home life isn’t going well, when I don’t have a job, when I don’t have that support system, when my housing is not where it should be, that those are the type of things that will contribute to me…overdosing…Just because…it would make me feel hopeless…I would stop caring so much about me, myself, or my life, you know? So, I wouldn’t be as mindful as to how much I’m taking or what I’m doing or putting myself in those type of situations as to how I could potentially overdose, you know? (ID102).

Social support networks were reported as being protective against overdose and return to illicit drug use post-release: “[H]aving a good support system, having a plan in place, and having positive individuals around you, you’ll have a better opportunity of not returning back to addiction than you didn’t have that at all” (ID101). Others spoke about the risk of returning to the community without any social support network, saying that “[i]f you go in the streets alone with nobody there to help you, of course you’re going to get high. And with the drugs out there nowadays it’s dangerous” (ID204). Duration of incarceration was also discussed as a potential risk factor for loss of social support networks and subsequent increased risk of overdose:

[W]hen people do a significant amount of time…maybe 18 months or more, sometimes they don’t make it to work…or they don’t have anybody outside to support them and all that stuff will…make them depressed so it’s almost like, ‘Why the hell am I gonna get out and do the right thing because I don’t have anything out there. I might as well keep getting high because that’s all I got’ (ID501).

Other participants described the importance of nonjudgmental community services, such as organizations that offered harm reduction education, syringe exchange services, and support for food insecurity:

[W]e have a place called [NONPROFIT 3] around here, and they help out a lot in the community, a lot…their goal is they don’t want addicts to share needles or share anything that they’re using. They want you to do everything clean. So, they give everything brand new…they also do a lot for community…There’s a town of [CITY 17] that’s nearby where I live and that town is so bad, we walk all over town, you see needles all over the town. So, they go around and clean the needles and…pick up all that trash…They also do food, like addicts can go there to their program and eat, they always have food for the addicts and…the homeless…I give them support a lot, because they really do support me. Actually, the way they treat people there, they’re loving people. They don’t judge nobody (ID307).

One individual summarized the importance of social support for people in recovery, saying “Support’s a huge thing, man…you can’t do it on your own…you need to do it for yourself, but you can’t do it by yourself” (ID304). Taken together, these quotes highlight the importance of social structures for preventing overdose among people recently released from jail, including stable housing and social support networks. The mental health status of people recently released from jail was reported as a key driver of overdose risk, with multiple participants describing how incarceration contributes to stress and adverse mental health states such as depression, which can lead to riskier drug use and harm.

### Demographics

Participants were asked to describe personal characteristics such as race, gender, ethnicity, and other background demographics that may contribute to overdose risk. Out of all personal characteristics, age and lived experience were identified as some of the most important factors that determine an individual’s risk of overdose in the post-release period. Young people who were recently released from jail were described as being at higher risk of overdose compared to older people, who often have more experience with drug use and personal experience with overdose. One participant explained that “A younger guy’s…going to think he can take the world and do what he used to do versus, somebody that maybe OD’d a couple of times and knows…what it’s like…[Y]ounger people…don’t have really experience as much with ODing” (ID603). Another reflected on his own experience as a young adult dealing with opioid addiction:

…when I was 18 years old, I wasn’t trying to listen to anything people were trying to tell me…So, I think being younger has a lot to do with it ‘cause you tend to find out the hard way, for lack of a better term, you tend to find out the hard way on your own because…you think you know it. You don’t want to listen to anybody. And I realize this because now looking back on everything I’ve been through…I wish I listened to those people…at 18 years old…everything they were talking about…overdosing and possibly dying, like all that happens (ID501).

Experience with overdose, whether personal or through peers, was described as protective against future overdose. One participant described learning about a friend who passed away from an overdose and sharing this news with others in jail:

I had an experience where I had a friend that was [incarcerated] with me for about eight months. And he was a good kid and he told me, ‘I’ll do the right thing. I’mma do the right thing.’ He went home and I wrote him a letter like a month later and his family mailed me his obituary. And that broke my heart. And I showed everybody that obituary and said this is what happens when you go home and you fuck up. And people were moved by that because that was a real reality check. A lot of people knew him you know. And they couldn’t believe it (ID301).

The above participant concluded: “And I said a lot of us got to wake up because this can happen to any one of us you know (ID301),” which spoke to a number of participants’ responses; despite the perception that younger age and limited lived experience with overdose contributed to increased overdose risk among participants, many also felt that overdose risk was indiscriminate in regard to demographic characteristics:

I don’t think race…Or anything that makes up an individual would contribute…I believe it all depends on the individual. Some people just don’t use their heads and they do too much, and they think they can do more than what they can, and they overdose…Sometimes, maybe the drugs are just too strong…Or a bad batch sometimes…but I don’t think that…somebody’s race or their background would be more of a reason why they would – or could overdose more so than anything else (ID102).

One participant summarized this perspective, saying “I think [overdose] can happen to anybody at any moment…any age, any race, it doesn’t pick and choose” (ID301). Taken together, these quotes illustrate how, although overdose is perceived to be ubiquitous, lived experience with overdose from either oneself or one’s peer networks can offer some degree of protection through shared knowledge.

### Economic and policy

Though economic and policy domains were discussed less explicitly in the interview guides than the other domains of the risk environment framework, participants still discussed economic and policy factors that contributed to risk of post-release overdose. For instance, lack of income generation during incarceration, post-release unemployment, and the financial challenges of addiction were described as barriers to long-term recovery and contributors to increased overdose risk in the post-release period:

…most of the time somebody that’s addicted…their lives are just shattered, their kids are somewhere else or [their] parents or their loved ones don’t talk to them, because they stole everything from them. So…they couldn’t hold a job because…they can’t show up, they’re not showing up for life. You can’t show up for life when you’re using any substance to the point where you’re abusing it (ID203).

Others discussed how incarceration itself lead to economic stagnation and instability, which lead to feelings of hopelessness and increased risk-taking behaviors, but work-release programs and other economic supports for people who have experienced incarceration can potentially reduce this risk:

…those last two years [of incarceration]…they prepare you to come home…they send you [to] the prerelease, where you’re working, they help you get a bank account. Putting money in the bank…so when your sentence is up you have something to fall back on. When you get released…you have a job you can keep, you have a bank account, you have money in the bank, you can get a place to live, they can help you get into a sober house, or whatever the case may be, so you have something to fall back on…And they want you to do all these programs, which is good, but then they send you to work for…four or five, six months, that’s not enough time to save up and get on your feet to the point where you’re going to be comfortable…I think the work release part should be…extended (ID501).

Participants supported the jail MOUD program and expansion of policies which allowed for access to MOUD in carceral settings, with one participant stating: “I think that the [MOUD] program is definitely beneficial, that the pros definitely outweigh the cons by a lot…I see more people that I know are clean due to the MAT being offered…in the jail system and the prison system” (ID202). Another participant shared that “I completely turned my life around on this medication. So, if it wasn’t for [the jail MOUD] program…I wouldn’t be able to be around right now… I started this medication in jail…I would never have taken it if they didn’t offer to me at that time while I was incarcerated” (ID304).

## Discussion

The continued overdose crisis and mass incarceration in the United States has led to disproportionate risk of overdose and drug-related death for people who have experienced incarceration [25–28]. The Risk Environment Framework provides a useful conceptual model for analyzing intersectional risks across multiple domains, with a focus on how individuals interact with their environments to both produce and reduce harm [10]. Although some studies have examined perceptions of overdose risk from the perspective of people who have experienced incarceration [15–18], these studies do not focus on people who received consistent MOUD while incarcerated, which is a population that has unique insights and lived experience with overdose risk upon release. Our study is novel in that it solicits the perspective of individuals who received MOUD during incarceration and analyzes the complex risk environment in the post-release period. Our findings demonstrate that formerly incarcerated individuals who received MOUD during their incarceration in Massachusetts jails have a high degree of knowledge of the risks inherent in the post-release environment and factors which may increase or decrease post-release overdose risk.

Participants reported that MOUD treatment during incarceration prevented loss of opioid tolerance, which was identified as one of the most common contributing factors to overdose risk. They identified the importance of establishing a therapeutic dose of MOUD while incarcerated and offering treatment for sufficient periods of time prior to release as protective factors against overdose. The converse was also true—when MOUD was only offered immediately prior to release or in insufficient doses for therapeutic effect, participants reported that protectiveness was mitigated. These results are consistent with prior research that demonstrated MOUD treatment during incarceration is protective against post-release overdose, therapeutic doses of MOUD are more protective than low doses, and underdosing of MOUD may be a risk factor for post-release return to use and overdose [5, 29–32]. Our findings also lend support to previous research which identified that interruptions in opioid use and forced withdrawals contribute to increased overdose risk [17]. MOUD provision during incarceration and subsequent linkage to MOUD treatment post-release is a key intervention to prevent overdose risk in justice-involved populations.

The findings support the Risk Environment Framework as a useful conceptual model for analyzing intersectional risks across multiple domains, with a focus on how individuals interact with their environments to both produce and reduce harm [10]. In the physical micro-environment, tolerance loss was reported to compound many other risk factors to produce risk of overdose, including personal drug use behaviors such as polysubstance use as well as macro-level factors such as adulteration of the illicit drug supply with fentanyl and exposure to drug activity when accessing treatment. Our findings lend support for interventions that target these risks, including education for people who are incarcerated around the dangers of polysubstance use and equipping them with basic harm reduction knowledge and tools (e.g., naloxone training and distribution) while incarcerated, as well as addressing environmental triggers for use in treatment neighborhoods and community investment and expansion of harm reduction programming such as syringe service programs, increased naloxone access, and “never use alone” hotlines [33–35]. Interventions that target macro-level factors such as contamination of the illicit drug supply include interventions in drug distribution routes and expansion of access to prescription opioids and MOUD, often referred to as access to “safe supply” [10, 36, 37]. Although participants in our study did not identify specific interventions that might reduce risk due to adulteration of the drug supply, it was reported as a major source of fear and risk for this population.

In the social micro-environment, the most important factors relating to overdose risk were social support and peer networks. Administration of naloxone, calling emergency medical services, and using opioids with another person present, rather than alone, were reported to be the most important ways that individuals can prevent overdose death. Overdose risk was discussed as indirectly prevented through mutual support and community building. As another example of peer-to-peer support preventing overdose, one participant described how building a relationship of trust between drug sellers and buyers is an important strategy to mitigate risks due to using alone and contamination in the illicit drug supply. Previous research has described how drug sellers are embedded in their communities and are often the most prominent source of harm reduction knowledge for drug buyers—making them important targets for public health interventions [38–40]. Our findings support interventions which empower peer networks and drug sellers to respond to overdoses, test drugs for adulterants, and share harm reduction knowledge.

The majority of participants reported that overdose risk was indiscriminate and could affect anyone regardless of sociodemographic factors, though younger age was identified as a risk for overdose due to lack of personal experience and decreased exposure to peers’ knowledge. Younger age is a known risk factor for overdose [41]; however, several national studies have identified sociodemographic factors which are associated with increased risk of overdose, including male sex and Black, Native American, or Alaskan Native race [42–44]. Women and Black and Native American/Alaskan Native individuals were underrepresented in our study, which may have limited our findings around sociodemographic factors and overdose risk. The perspective that overdose was ubiquitous is nonetheless an important insight as it reflects the widespread fear towards overdose among participants.

Social determinants of health such as lack of sufficient housing and poor mental health care accessibility were also reported to be key drivers of overdose risk that may compound other risks. These results support risk reduction strategies that target and empower peer networks to intervene during overdoses and share harm reduction knowledge, such as education about and provision of naloxone for people who are recently released from incarceration, as well as systemic level investments in housing and mental health care [45–47].

This study provides novel insights into the perceptions of post-release overdose risk from people with OUD who have experienced incarceration in Massachusetts jails and received MOUD while incarcerated. Deductive validation of the Risk Environment Framework through examination of the lived experience of participants who are most directly impacted by these risks is a strength of this study, especially as peer-to-peer harm reduction knowledge transmission was identified as a key protective strategy against overdose. Another strength of this study is that data were captured during a high-risk period for overdose death in Massachusetts, with overdose death rates increasing by 2.5% from 2021 to 2022 and an almost 10% rise in the overdose death rate compared to pre-COVID-19 pandemic rates [48]. By capturing patient perspectives during this critical period of risk, we can better identify interventions that target and mitigate overdose-related harm. Our sample was majority White and male, which is a limitation of this study. Another limitation of this study is that the instrument did not specifically ask questions about policy and economic risk factors or interventions to prevent overdose risk, which meant that many participants did not discuss these aspects of the Risk Environment Framework. However, participants’ insights often touched on broader systemic factors and related them to the physical and social factors that increase and prevent overdose risk. Future studies should directly solicit perceptions from people with lived experience of OUD and incarceration about economic and political risk factors for overdose so that these valuable perspectives might inform systemic-level interventions.

## Conclusions

Participants with lived experience of OUD, incarceration, and MOUD treatment during incarceration reported a high degree of knowledge of the risks of the post-release environment and identified critical gaps in treatment access and healthcare systems. Participants reported micro-level factors which increase overdose risk, including tolerance loss during incarceration, underdosing of MOUD, and lack of social support upon release. Early MOUD induction during incarceration with continued treatment upon release, peer-driven harm reduction, and community reentry support were reported to be protective against overdose risk. On the macro-level, participants described how the toxic illicit drug supply and lack of accessible housing and mental healthcare predisposed individuals to greater risk of overdose. Overdose was reported to be ubiquitous and a widespread source of fear in this population. Public health interventions that seek to reduce overdose risk in this population should engage individuals across multiple domains of the risk environment framework.

## Data Availability

The dataset analyzed during the current study is not publicly available to protect participants’ privacy but is available from the corresponding author on reasonable request.

## Abbreviations

MOUD: Medications for opioid use disorder
MA: Massachusetts
JCOIN: Justice Community Opioid Innovation Network
OUD: Opioid use disorder
XR: Extended-release
